# Targeted Analysis of Plasma Polar Metabolites in Postmenopausal Depression

**DOI:** 10.3390/metabo14050286

**Published:** 2024-05-16

**Authors:** Maria Fernanda Naufel, Amanda Paula Pedroso, Adriana Pereira de Souza, Valter Tadeu Boldarine, Lila Missae Oyama, Edson Guimarães Lo Turco, Helena Hachul, Eliane Beraldi Ribeiro, Mônica Marques Telles

**Affiliations:** 1 Department of Physiology, Universidade Federal de São Paulo (UNIFESP-EPM), Rua Botucatu 862, Vila Clementino, São Paulo 04023-062, SP, Brazil; amandapedroso.bio@hotmail.com (A.P.P.); adrikasouza@gmail.com (A.P.d.S.); valtertadeuboldarine@gmail.com (V.T.B.); lmoyama@unifesp.br (L.M.O.); mmtelles@unifesp.br (M.M.T.); 2 Department of Surgery, UNIFESP-EPM, São Paulo 04023-062, SP, Brazil; edsonlt@gmail.com; 3 Department of Psychobiology, UNIFESP-EPM, São Paulo 04023-062, SP, Brazil; helenahachul@gmail.com; 4 Department Gynaecology, UNIFESP-EPM, São Paulo 04023-062, SP, Brazil

**Keywords:** polar metabolites, postmenopausal women, premenopausal women, depression, mood disorders, amino acids

## Abstract

Depression will be the disease with the highest incidence worldwide by 2030. Data indicate that postmenopausal women have a higher incidence of mood disorders, and this high vulnerability seems to be related to hormonal changes and weight gain. Although research evaluating the profile of metabolites in mood disorders is advancing, further research, maintaining consistent methodology, is necessary to reach a consensus. Therefore, the objective of the present study was to carry out an exploratory analysis of the plasma polar metabolites of pre- and postmenopausal women to explore whether the profile is affected by depression. The plasma analysis of 50 polar metabolites was carried out in a total of 67 postmenopausal women, aged between 50 and 65 years, either without depression (n = 25) or with depression symptoms (n = 42), which had spontaneous onset of menopause and were not in use of hormone replacement therapy, insulin, or antidepressants; and in 42 healthy premenopausal women (21 without depression and 21 with depression symptoms), aged between 40 and 50 years and who were not in use of contraceptives, insulin, or antidepressants. Ten metabolites were significantly affected by depression symptoms postmenopause, including adenosine (FDR = 3.778 × 10^−14^), guanosine (FDR = 3.001 × 10^−14^), proline (FDR = 1.430 × 10^−6^), citrulline (FDR = 0.0001), lysine (FDR = 0.0004), and carnitine (FDR = 0.0331), which were down-regulated, and dimethylglycine (FDR = 0.0022), glutathione (FDR = 0.0048), creatine (FDR = 0.0286), and methionine (FDR = 0.0484) that were up-regulated. In premenopausal women with depression, oxidized glutathione (FDR = 0.0137) was down-regulated, and dimethylglycine (FDR = 0.0406) and 4-hydroxyproline (FDR = 0.0433) were up-regulated. The present study provided new data concerning the consequences of depression on plasma polar metabolites before and after the establishment of menopause. The results demonstrated that the postmenopausal condition presented more alterations than the premenopausal period and may indicate future measures to treat the disturbances involved in both menopause and depression.

## 1. Introduction

Depression negatively impacts the quality of life. It is associated with attention and memory deficits, decreased productivity, sleep disorders, weight gain or loss induced by changes in eating behavior, alcohol and drug abuse, and increased suicidal thoughts [1,2]. It also increases the risk of chronic diseases, especially cardiometabolic diseases, metabolic syndrome, type 2 diabetes, heart disease, and stroke, which are among the diseases with the highest mortality in the world [3,4,5].

In addition to personal, family, and social losses, mental disorders also have an economic and health system impact. Estimates indicate that depression and anxiety can cause losses of more than 2.5 trillion dollars per year worldwide due to productivity loss [6]. The WHO assessed that depression will be the disease with the highest incidence worldwide by 2030 [7].

Despite affecting individuals of both sexes and all ages, depression is more evident in adult women [7,8]. Data from the literature indicate that postmenopausal (PM) women have a higher incidence of mood disorders [9,10,11], with susceptibility to the manifestation of the binomial depression/obesity increased when compared to premenopausal women [12,13]. The high vulnerability to this neuropsychiatric disorder seems to be related to hormonal changes and weight gain [14], and the prevalence of obesity and risk of metabolic changes in PM can affect up to 50% of women between 50 and 59 years old [15].

Our group showed a significant increase in depression symptoms in women in late PM when compared to premenopausal women [12]. Furthermore, using an animal model, we showed that ovariectomized rats had a higher level of anxiety [16] and that the deficiency of ovarian hormones is associated with obesity-induced depressive-like behavior in these animals [17].

Metabolomics analysis provides insight into the metabolites of a system [18,19]. Metabolomics is, today, one of the most essential “omic” methodologies, allowing for the evolution from merely observational studies to a detailed understanding of the mechanisms triggering the studied pathology [18,20,21].

The use of “omics” techniques in research in the field of obesity and mood changes has been identified as extremely promising. Analysis of metabolites can contribute to understanding the processes involved in the progression of these diseases and enable the identification of biomarkers. Furthermore, this analysis technique makes it possible to develop diagnostic, preventive and/or early treatment methods for several disorders [22].

Polar metabolite analysis involves the study of small polar molecules like amino acids and polyamines. These molecules are usually involved in primary metabolism and essential metabolic pathways necessary for survival. Polar metabolites have been recognized as biomarkers for a range of health conditions such as cancers [23], diabetes [24], infarction [25], and Alzheimer’s disease [26], suggesting that they may serve as biomarkers for various diseases.

Studies suggest that depression causes changes in plasma metabolites, such as phosphatidylcholine and sphingomyelin [27]. A meta-analysis study observed that women in PM had high levels of glutamate and phosphatidylcholines 36:1 and 38:3 and low levels of tryptophan [28]. These data indicate that the assessment of metabolic changes in depression is relevant.

Although research evaluating the profile of metabolites in mood disorders is advancing, further research, maintaining consistent methodology, is necessary to reach a consensus. Therefore, the objective of the present study was to carry out an exploratory analysis of the plasma polar metabolites of pre- and postmenopausal women to explore whether the profile is affected by depression.

## 2. Materials and Methods

The present study was approved by the Ethics Committee of the Universidade Federal de São Paulo (CEP# 921.394/2014 and 0624/2019). The information obtained was used only for scientific purposes, preserving confidentiality regarding the identity of the participants. The procedures were conducted in accordance with the Declaration of Helsinki and the ethical standards of the Brazilian Health Council (Resolution 466/2012) [29]. All subjects signed the informed consent before their participation in the study.

Postmenopausal women with at least 12 months of amenorrhea and serum FSH concentration > 30 IU/mL were included. The selected women were aged between 50 and 65 years, had spontaneous onset of menopause and were not in use of hormone replacement therapy, insulin, or antidepressants. The premenopausal group included women in the reproductive stage (regular menstruation), healthy, aged between 40 and 50 years, with FSH levels < 30 IU/mL, who were not in use of contraceptives, insulin, or antidepressants. Premenopausal women were matched by body mass index (BMI) to the postmenopausal group.

The recruitment of volunteers was carried out through the distribution of pamphlets, and through the Press Office of UNIFESP, which was publicized through electronic and printed media and in radio advertisements.

Of the 102 postmenopausal women initially included in the study, 35 were excluded due to antidepressant use (n = 17), hypothyroidism (n = 15), or absence of blood sample for metabolomic analysis (n = 03). Thus, the final sample consisted of 67 postmenopausal women (25 without depression and 42 with depression symptoms).

Regarding the premenopausal women, baseline anthropometric, clinical, and biochemical analyses were collected from 45 participants. However, 3 were excluded due to the diagnosis of hypothyroidism (n = 2) or absence of blood sample for metabolomic analysis (n = 1). The final sample of premenopausal women consisted of 42 volunteers (21 without depression and 21 with depression symptoms).

The procedures executed for this study have been previously described [12]. Briefly, participants were instructed to present themselves with 12 h of fasting beforehand on the scheduled day when anthropometric assessment was performed; the Beck Depression Inventory was applied, and blood was withdrawn for FSH analysis. Saliva was collected for cortisol analysis. The method used for collecting saliva samples was via Sallivette^®^ swabs (Sarstedt AG & Co., Numbrecht, Germany). After collection, the samples were centrifuged and analyzed immediately for cortisol levels using an ELISA kit from LDN—Labor Diagnostika Nord (Nordhorn, Germany). The coefficient of variation for the test was 8.1%.

Data on the presence of diabetes, hypertension, and exercise practice were self-reported. The participants were asked to answer “yes” or “no” whether they had been previously diagnosed with diabetes or hypertension and whether they routinely engaged in exercise.

### 2.1. Anthropometric Assessment

Body composition was determined by direct segmental multi-frequency bioelectrical bioimpedance analysis (InBody230^®^, Seoul, Republic of Korea), with volunteers wearing light clothing and being barefoot. The following data were obtained: body weight, body mass index, body fat percentage (BDP), muscle mass in kilograms (MM), basal metabolic rate (BMR), fat-free mass, and waist-to-hip ratio (WHR). A study showed that the data provided by the InBody 230^®^ system had good reliability compared to the Dexa and BodPod methods [30].

### 2.2. Assessment of Depression Status

Beck Depression Inventory (BDI) is a self-administered questionnaire, consisting of 21 multiple-choice items, each with four alternatives, and an increasing score (0–3) according to the severity of the symptoms. The patient ticks the answer that corresponds to how they felt in the last week, including the day of the exam. The sum of the scores supports the classification of depression intensity levels, as follows: none (0–9), mild (10–16), moderate (17–29), or severe (30–63) [31]. The results were validated for the Brazilian population in 2001 [32].

### 2.3. Experimental Groups

The following groups were evaluated:

- Pre ND: Premenopausal women, without depression symptoms (n = 21).
- Pre D: Premenopausal women with depression symptoms (n = 21).
- Post ND: Postmenopausal women without depression symptoms (n = 25).
- Post D: Postmenopausal women with depression symptoms (n = 42).

### 2.4. Polar Metabolites Analysis

Extraction

Aliquots of 50 µL of plasma were added into a 96-deep well plate cell to which 1 µL of thiol-derivatization solution (200 mM N-ethylmaleimide (NEM) + 2 mM citric acid) was added. Subsequently, 200 µL of precipitation solution (acetonitrile/isopropanol (7:3, *v*/*v*) + 1% formic acid + isotopically labeled internal standards) were added. After vortex agitation for 10 min and refrigeration at −20 °C for 10 min, the samples were centrifuged (14,000 rpm for 10 min at 4 °C).

The following 50 metabolites were analyzed: cysteine, asparagine, aspartic acid, serine, alanine, 4−hydroxyproline, glycine, glutamine, threonine, dimethylglycine, glutamic acid, citrulline, proline, ornithine, 2−aminobutyric acid, lysine, histidine, adenosine monophosphate, arginine, creatine, hypoxanthine, choline, uridine, valine, creatinine, carnitine, methionine, guanosine, pantothenic acid, tyrosine, adenosine, asymmetric dimethylarginine, isoleucine, leucine, phenylalanine, kynurenine, acetyl−carnitine, tryptophan, isocitric acid, lactic acid, uric acid, succinic acid, fumaric acid, TMAO, glutathione, taurine, oxidized glutathione, cysteine, kynurenic acid, 2−ketoglutaric acid.

### 2.5. Chromatographic Analysis Coupled to Mass Spectrometry (LC-MS/MS)

The extracts were diluted in isopropanol/water (3:7, *v*/*v*). The chromatographic separation was carried out in a Nexera CL chromatographic system (model LCMS-8060CL, Shimadzu Corporation, Kyoto, Japan). Five microliters of the diluted samples were injected into the column (Discovery^®^ model HS-F5-3, PFPP resin (3 µm, 150 mm × 2.1 mm, Supelco), maintained at 40 °C, and run at 0.25 mL/min for 25 min. The mobile phases consisted of (A) water + 0.1% formic acid and (B) acetonitrile + 0.1% formic acid. Phase A gradient was from 100 to 5% in 15 min, maintained 5% for 5 min, and then changed to 100% at minute 20.1.

The mass spectrometer with a triple-quadrupole analyzer (model LCMS-8060CL, Shimadzu Corporation, Kyoto, Japan) operated with electrospray ionization source in positive and negative modes. Detections were made in multiple reactions monitoring (MRM) mode.

Data acquisitions were carried out using the LabSolutions software (Shimadzu, Kyoto, Japan) while the quantification of metabolites was carried out using the Insight 3.7 software (Shimadzu).

The online software MetaboAnalyst 3.0 (http://metaboanalyst.ca, accessed on 2 February 2024) was used for analysis of metabolomics data. Normalization by sum, log transformation, and auto scaling were applied.

### 2.6. Statistical Analysis

A descriptive analysis of all parameters was carried out. The Shapiro–Wilks normality test was applied. Parametric data were analyzed by the Student’s *t* test and non-parametric data by the Mann–Whitney test, while qualitative variables were compared by the chi-squared or Fisher’s exact tests.

Multivariate partial least square discriminant analysis (PLS−DA) models were built to recognize the metabolites with the most significant discriminatory effect between the groups of women with and without depression symptoms, with the highest projection power.

To determine the model quality, we used the values of R2 and Q2, found through cross-validation. The R2 parameter represents how much variation within the set was explained by the model’s components, while the Q2 parameter indicates the model’s projection power. Through PLS-DA, it was also possible to construct the importance of the variable (VIP), which we used to identify metabolites with values higher than 1.0 between groups in the component with the highest projection power.

The statistical analysis was performed by Student’s *t*-test and corrected by the false discovery test (FDR). The significance level adopted was FDR < 0.05.

Fold-change (FC) and receiver operating characteristic (ROC) curves were constructed to assess the performance of potential discriminants [33]. Across the ROC curve, we evaluated the area under the curve (AUC). AUC < 0.5 indicates that the test has no diagnostic value, AUC 0.8–0.9 indicates good accuracy, and AUC > 0.9 suggests that the diagnostic test has high accuracy [34].

## 3. Results

### 3.1. Postmenopausal Women

The postmenopausal women with and without depression presented statistically similar values of age, years after menopause, BMI, cortisol levels, BFP, and muscle mass. FSH levels and self-reported hypertension incidence were significantly higher in postmenopausal women with depression symptoms. Cortisol levels were similar between the groups. The self-reported data on the presence of diabetes, dyslipidemia, and exercise were similar between the groups. The group with depression symptoms had higher BDI scores. No covariant/confounders were considered for these analyses (Table 1).

The analysis of polar metabolites included the 50 compounds shown in Table 2. Ten metabolites were significantly affected by depression variations. Adenosine, guanosine, proline, citrulline, lysine, and carnitine were down-regulated while dimethylglycine, glutathione, creatine, and methionine were up-regulated in postmenopausal women with depression symptoms (Table 3).

**Figure 1:**
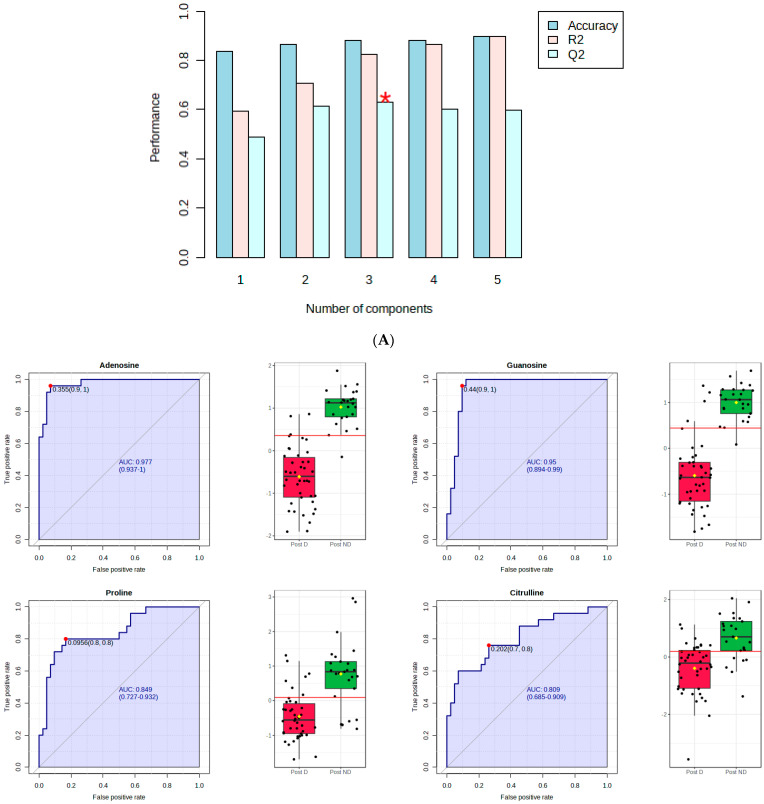

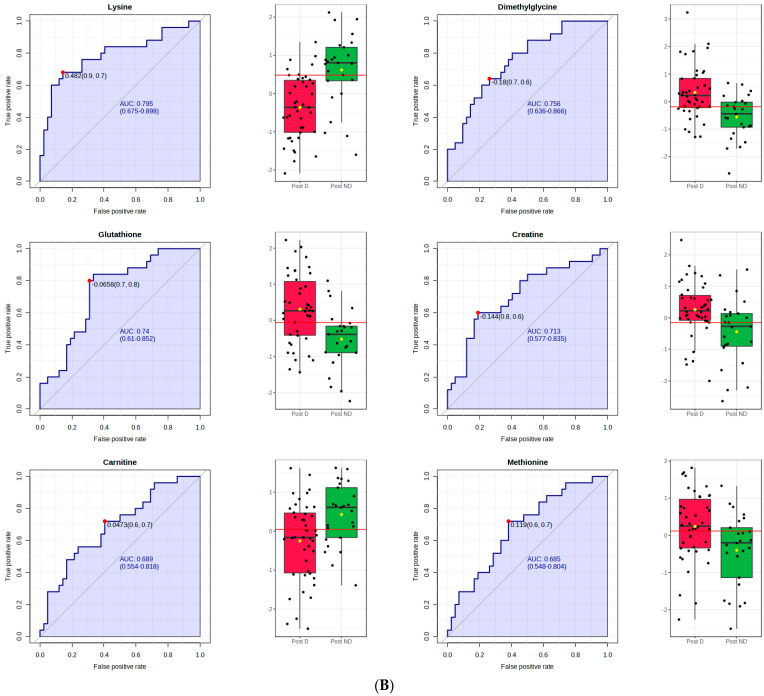
PLS–DA and receiver operating characteristic (ROC) analysis of polar metabolites significantly affected by depression in postmenopausal women. (**A**) component 3 of the PLS–DA model; (**B**) the ROC curve analysis of the significant polar metabolites.

Figure 1A shows that component 3 of the PLS-DA model had Q2 of 0.62, R2 of 0.82, and an accuracy of 0.89. The red star indicates the best classifier.

Figure 1B shows the ROC curve analysis of the significant polar metabolites. Across the ROC curve, we evaluated the area under the curve (AUC). AUC < 0.5 indicates that the test has no diagnostic value, AUC 0.8–0.9 indicates good accuracy, and AUC > 0.9 suggests that the diagnostic test has high accuracy [33]. Red indicates postmenopausal women with depression and green indicates postmenopausal women without depression. A horizontal line is in red indicating the optimal cutoff.

### 3.2. Premenopausal Women

The premenopausal women with and without depression presented statistically similar values of age, BMI, BFP, and FSH. Muscle mass was significantly higher in premenopausal women with depression, while self-reported exercise was significantly higher in the group without depression. The cortisol levels, self−reported data on the presence of diabetes, hypertension, and dyslipidemia were similar between the groups. The group with depression symptoms had higher scores of BDI. No covariant/confounders were considered for these analyses (Table 4).

The analysis of polar metabolites in the premenopausal women group included the 50 compounds shown in Table 5. Three metabolites were significantly affected by depression. Oxidized glutathione was down-regulated, while dimethylglycine and 4-hydroxyproline were up-regulated in premenopausal women with depression symptoms (Table 6).

**Figure 2:**
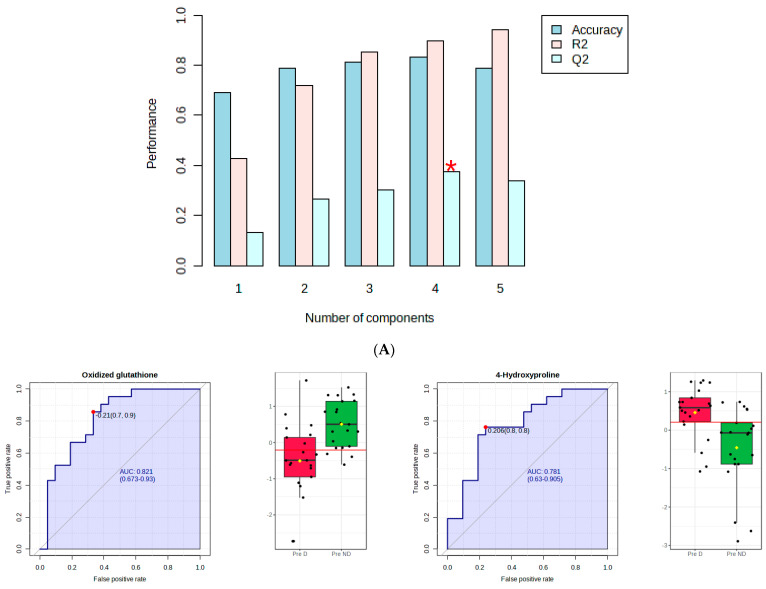

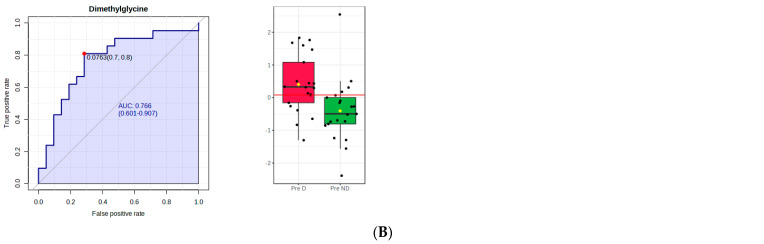
PLS−DA and receiver operating characteristic (ROC) analysis of polar metabolites significantly affected by depression in premenopausal women. (**A**) component 4 of the PLS−DA model; (**B**) the ROC curve analysis of the significant polar metabolites.

Figure 2A shows that component 4 of the PLS-DA model had Q2 of 0.38, R2 of 0.89, and an accuracy of 0.83. The red star indicates the best classifier.

Figure 2B shows the ROC curve analysis of the significant polar metabolites. Across the ROC curve, we evaluated the area under the curve (AUC). AUC < 0.5 indicates that the test has no diagnostic value, AUC 0.8–0.9 indicates good accuracy, and AUC > 0.9 suggests that the diagnostic test has high accuracy [33]. Red indicated premenopausal women with depression and green indicated premenopausal women without depression. A horizontal line is in red indicating the optimal cutoff.

## 4. Discussion

We have previously found that postmenopausal women had a higher incidence of depression symptoms in comparison to premenopausal women [12]. Moreover, it has been shown that menopause is associated with changes in plasma metabolite levels, such as phosphatidylcholines, sphingomyelins, and amino acids [35]. The present article aimed at analyzing how the profile of plasma polar metabolites associates with depression symptoms in post- and premenopausal women.

In postmenopausal women with depression, we observed that the levels of ten plasma polar metabolites differed significantly from those in postmenopausal women without depression. The primary metabolites with the most relevant differences between these two groups were adenosine and guanosine, showing lower levels in postmenopausal women with depression symptoms.

Adenosine is an endogenous autacoid present in all mammalian tissues. It is crucial in regulating various physiological functions and is the primary component of ATP. Moreover, it is involved in multiple pathologies, including cancer and inflammatory and neurological disorders. In the central nervous system (CNS), adenosine controls neuronal excitability, synaptic plasticity, and neuron degeneration. It even plays a crucial role in the modulation of astrocytic and microglial cells [36].

To the best of our knowledge, this is the first study to find low levels of plasma adenosine in postmenopausal women with depression. Based on the available evidence, it seems that the reduction in adenosine synthesis plays a crucial role in the development of symptoms associated with major depressive disorder (MDD). Moreover, increasing adenosine levels, through pharmacological or non−pharmacological therapy, had a positive effect on depressive and anxious symptoms [37].

Adenosine interacts with four subtypes of receptors, A_1_R, A_2A_R, A_2B_R, and A_3_R, each with distinct functions. The receptors A_1_R and A_2A_R are the most studied in depression and other related pathologies. The benefits of adenosine, with its antidepressant and anxiolytic effects, are mainly related to the activation of the A_1_R, as this receptor has shown to play a significant role in regulating depressive and anxious behaviors. Concerning A_2A_R, evidence has suggested that increased A_2A_R signaling, which can be triggered by exposure to stress, has negative effects on mood and can lead to depression. At the same time, the treatment with A_2A_R antagonists has been shown to prevent or reverse depressive symptoms [37,38].

According to an experimental study, the expression of A_1_R, A_2A_R, and A_3_R were decreased in the brain of female rats, 3 months after ovariectomy, suggesting that hormones produced by the ovaries may play a role in regulating adenosine receptor expression in the brain [39].

Although there is evidence to support that adenosine and the adenosinergic system play a role in the pathogenesis of depression, and that adenosine supplementation and the development of medications to target this system may be an effective approach to treating the disorder mainly in postmenopausal depression, in which we demonstrated lower levels of adenosine, more studies are necessary to determine its therapeutic potential.

Guanosine also differed between the postmenopausal groups, being significantly lower in the group with depression. Guanosine is a purine nucleoside believed to have protective properties for the nervous system. It is released in the brain during physiological responses and disease conditions, decreasing neuroinflammation, oxidative stress, and excitotoxicity. Moreover, it presents trophic effects on both neuronal and glial cells. Guanosine is protective for CNS diseases, such as ischemic stroke, Alzheimer’s disease, Parkinson’s disease, and depression. The neurobiological properties of this polar metabolite are associated with activating multiple intracellular signaling pathways and interacting with the adenosinergic system [40].

There is solid evidence that imbalances in the blood levels of purines, such as guanosine and adenosine, are linked to depressive symptoms [41,42]. Additionally, preclinical research has shown that guanosine administration can produce antidepressant-like effects, further supporting its potential as a treatment for depression [41,43]. The potential of guanosine to act as an antidepressant may involve the modulation of adenosine receptors [41].

As adenosine and guanosine are expressively decreased in the plasma of postmenopausal women with depression, we suggest that future studies should focus on the biochemical analyses and supplementation of these purines to see whether they are able to prevent or reduce depression symptoms in postmenopausal women.

The metabolite proline was also significantly lower in the plasma of postmenopausal women with depression symptoms than in the ones not presenting those symptoms. Conversely, in the premenopausal women with depression symptoms, 4-hydroxyproline was higher when compared to premenopausal women without depression symptoms.

Proline works alongside arginine, glutamine, and leucine to boost protein synthesis in cells and tissues. This metabolite is the precursor of 4-hydroxyproline, and both are essential components of collagen proteins [44]. The collagen-derived dipeptide prolyl-hydroxyproline, which is composed of proline and hydroxyproline residues, was detected in the cerebrospinal fluid, increased dopamine levels in the prefrontal cortex, and suppressed depression-like behaviors when administered orally to mice. However, the isolated oral administration of either proline or hydroxyproline showed no effect [45].

We could not find studies analyzing proline/hydroxyproline in women in the context of postmenopausal depression. In middle-aged (27–66 years of age) male and female volunteers with MDD, higher plasma proline levels have been found, in comparison to non-depressed individuals [46]. However, the MDD subjects analyzed in the study were in use of antidepressants, unlike the present subjects. In another recent report analyzing individuals of both genders (21–61 years of age), serum proline levels were up-regulated in the group diagnosed with depression, when compared to the healthy controls. This study also demonstrated higher proline levels in rats submitted to chronic mild unpredictable stress [47]. When the urinary concentrations of proline and hydroxyproline were analyzed in patients diagnosed with depression, anxiety, and stress (mean age of 34 years, both genders), the results showed that anxiety was positively correlated to proline, while hydroxyproline was weakly associated with stress. None of these two metabolites showed an association with depression symptoms. The proline levels were significantly higher in females when compared to the male group [48].

In ovariectomized mice, oral proline administration reduced body weight and luteinizing hormone levels, increased estradiol and alkaline phosphatase levels, and improved bone mineral density. The authors suggested proline as a promising option for menopause treatment [49].

As studies analyzing the association between mood disorders and proline or 4-hydroxyproline levels are still contradictory, and no study examining the levels in females with depression at the different stages of reproductive life has been found, more research is necessary to conclude the relationship of these two metabolites with depression symptoms in pre- and postmenopausal women.

Citrulline was significantly lower in postmenopausal women with depression. Citrulline is a non−essential amino acid; it is a precursor for L−arginine synthesis and an important component of the urea cycle. Moreover, citrulline and arginine are the precursors of nitric oxide (NO), a potent vasodilator that reduces blood pressure and improves oxygenation [50]. Postmenopausal women have high rates of endothelial dysfunction [51]. L−citrulline supplementation in postmenopausal hypertensive women increased L-arginine levels and ameliorated blood pressure and endothelial function [52].

A recent meta-analysis revealed that arginine and citrulline serum concentrations were significantly lower in MDD patients in comparison to healthy controls [53]. When the concentrations of L−arginine and L−citrulline were assessed in 35 unmedicated physically healthy patients with major depression and compared to 36 healthy controls, individuals with major depression presented significantly lower levels of L-arginine and L−citrulline.

The authors suggest that this reduction in the amino acid levels may be a potential reason for the decrease in NO observed in major depression patients. The reduction in NO concentrations, in turn, could lead to endothelial dysfunction and contribute to the increased cardiovascular risk associated with major depression [54]. We could not find studies evaluating the association of citrulline, and depression in post- or premenopausal women were found. The present findings indicate that citrulline deficiency postmenopause may contribute to the development of depression symptoms.

Lysine is an essential amino acid presently found decreased in the postmenopausal women with depression symptoms. L−lysine has been shown to act as an antagonist of serotonergic 5−HT 4 receptors, having a beneficial effect on the anxiety induced by stress in rats [55]. An anxiolytic effect has been found in humans, after its administration in combination with L−arginine [56]. No studies associating lysine with depression in middle-age women were found; thus, more studies are necessary to elucidate this association.

Dimethylglycine (DMG), a glycine derivative, was significantly higher in the plasma of both the premenopausal and the postmenopausal women with depression, when compared to the ones without depression. Experimental research has shown that DMG alone displayed an antidepressant-like effect in mice as it reduced the immobility in the forced swim test. The authors suggested that, as DMG is a nutritional supplement regarded as safe and non−toxic, it might be used as an adjunct to treat depression [57]. We were not able to find human studies evaluating the role of DMG in depression or menopause. Although the present results may indicate that stimulation of DMG production may be an endogenous mechanism to fight depression, further studies are necessary to ascertain this aspect.

While glutathione levels were significantly higher in postmenopausal women with depressive symptoms, when comparing to postmenopausal women without depression, among premenopausal women with depression, the levels of oxidized glutathione were significantly lower than in the group without depression.

The tripeptide glutathione (GSH), which is made of cysteine, glycine, and glutamic acid, exists in two forms: the reduced form (GSH) and the oxidized form (GSSG). In a pro−oxidant environment, two GSH molecules combine through a disulfide bond to form GSSG, while in antioxidant conditions, GSSG is transformed into GSH. This conversion is an aspect of the body’s antioxidant defense system, which helps prevent oxidative stress caused by accumulating reactive oxygen species (ROS). As GSH plays a crucial role in protecting the cells, including in the brain, from damage caused by ROS, low GSH levels render them more vulnerable to oxidative stress [58,59].

Previous research has indicated that patients with MDD experience higher levels of inflammation markers and oxidative stress when compared to healthy controls [60]. When analyzing GSH levels in the prefrontal cortex of postmortem patients diagnosed with MDD, researchers observed lower levels of the metabolite when compared to healthy controls [58]. A recent study has assessed in vivo GSH levels in the prefrontal cortex by magnetic resonance, finding them to be increased in patients with current MDD, when compared to those with past MDD and healthy controls [61]. Decreased levels of GSH were found in the occipital region of patients with anhedonia compared to age− and sex-matched controls [62]. However, when GSH and GSSG were analyzed in blood samples of unmedicated MDD patients, researchers found no significant difference compared to healthy controls [60].

There is still a lot of uncertainty surrounding the link between depression and glutathione or oxidized glutathione levels based on current studies. Additionally, there has been no research conducted on the levels of these two metabolites in females with depression during different stages of reproductive life, making it challenging to conclude their relationship with depression symptoms in pre- and postmenopausal women. Therefore, more research is needed to obtain a clearer understanding of the correlation between glutathione levels and depression symptoms in women.

Nevertheless, the significantly increased blood levels of glutathione in postmenopausal women with depression might indicate a physiologic up-regulation in GSH concentrations in response to a higher inflammation and oxidative stress of depression. In contrast, the low oxidized glutathione levels, found in premenopausal women with depression, may derive from a low production from GSH, a reaction important for antioxidant defense, and hence, indicate an ineffectiveness of the body at suppressing oxidative damage.

There are some limitations related to our study. The current study’s cross-sectional design implies that an assessment of temporality cannot be established. Additionally, since we sampled specifically middle-aged pre- and postmenopausal women, an inference that the findings may apply to the general population may not be made.

Despite its limitations, our study is the first to observe the association of various polar metabolites and depression symptoms in pre- and postmenopausal women—as far as we are aware—which paves the way for future research into potential biomarkers for depression.

## 5. Conclusions

The present study provided new data concerning the association of depression and plasma polar metabolites before and after the establishment of menopause. The results demonstrated that the postmenopausal condition presented more alterations than the premenopausal period and may indicate future measures to treat the disturbances involved in both menopause and depression.

## Figures and Tables

**Table 1:** Clinical and anthropometric parameters in postmenopausal women with or without depression.

	Post ND (25)	Post D (42)	*p*
Age (years) ^a^	55.9 ± 0.9	57.2 ± 0.6	0.221
Years after menopause ^a^	7.6 ± 1.1	8.4 ± 0.95	0.549
BMI (kg/m^2^) ^a^	28.0 ± 1.0	28.3 ± 0.7	0.799
FSH (mUI/dL) ^a^	65.3 ± 5.6	79.8 ± 3.9	0.036
Cortisol (ng/dL) ^b^	5.2 ± 0.4	8.1 ± 1.2	0.401
BFP ^a^	39.0 ± 1.4	39.5 ± 1.2	0.816
Muscle Mass (kg) ^a^	27.7 ± 1.9	28.7 ± 1.5	0.673
BDI ^a^	5.7 ± 0.6	19.1 ± 1.2	**<0.001**
Diabetes ^c^	8.0%	16.7%	0.314
Hypertension ^c^	8.0%	35.7%	0.012
Exercise ^c^	40.0%	57.1%	0.174

BMI: Post ND: postmenopausal women without depression; Post D: postmenopausal with depression. ^a^ Means ± SE, analyzed by Student’s *t* test. ^b^ Median (minimum–maximum), analyzed by Mann–Whitney U test. ^c^ Percentages analyzed by chi-squared test. BMI: body mass index; BFP: percentage body fat; BDI: Beck Depression Inventory score. Bold values indicate statistical significance.

**Table 2:** Fold-change and variable importance on projection (VIP) of plasma polar metabolites in postmenopausal women.

	Fold-Change (D/ND)	VIP
Proline	0.3988 *	1.8235
Guanosine	0.4174 *	2.3990
Adenosine	0.4575 *	2.4560
Hypoxanthine	1.6896	0.8120
4−Hydroxyproline	1.6838	0.6588
Lysine	0.6382 *	1.6433
2−Aminobutyric acid	1.5059	1.0236
Adenosine monophosphate	1.5019	0.4053
Dimethylglycine	1.4936 *	1.3144
Citrulline	0.6748 *	1.5789
Cystine	1.4698	0.3894
Creatine	1.3278 *	1.0478
Pantothenic acid	0.7708	0.8966
Aspartic acid	1.2737	1.1528
Isocitric acid	1.2533	0.9406
Carnitine	0.8020 *	1.2326
Fumaric acid	1.2419	0.7060
Glutamic acid	1.2342	1.0623
Histidine	0.8436	0.8997
Methionine	1.1709 *	1.0852
Glycine	1.1527	0.5705
Kynurenic Acid	0.8698	0.4935
Arginine	1.1479	0.9366
Succinic acid	0.8748	0.8084
Uridine	1.1425	0.4721
Oxidized glutathione	0.8785	0.6176
Glutathione	1.1354 *	1.2642
Valine	0.8827	0.8552
Isoleucine	1.1221	0.7252
Lactic acid	0.8914	0.6723
Glutamine	1.1200	0.9857
Ornitine	0.8985	0.7312
Phenylalanine	1.1102	0.8767
Tyrosine	1.1094	0.7783
Kynurenine	1.1006	0.7979
Serine	1.0865	0.5120
Asymmetric dimethylarginine	1.0781	0.6698
Asparagine	1.0746	0.4758
Tryptophan	1.0737	0.7294
Alanine	1.0694	0.6352
Cysteine	0.9352	0.6616
Choline	1.0637	0.6512
Creatinine	1.0588	0.6533
Uric acid	1.0580	0.7031
Threonine	1.0480	0.5508
2−Ketoglutaric Acid	0.9708	1.2005
Acetylcarnitine	1.0167	0.5066
Leucine	1.0074	0.6670
TMAO	0.9944	0.4392
Taurine	0.9964	0.6612

D: postmenopausal women with depression symptoms; ND: postmenopausal women without depression symptoms. * FDR < 0.05.

**Table 3:** Plasma polar metabolites significantly affected in postmenopausal depression.

Metabolite Name	Fold-Change (D/ND)	FDR	VIP
Adenosine	0.4575	3.778 × 10^−14^	2.4560
Guanosine	0.4174	3.001 × 10^−13^	2.3990
Proline	0.3988	1.430 × 10^−6^	1.8235
Citrulline	0.6748	0.0001	1.5789
Lysine	0.6382	0.0004	1.6433
Dimethylglycine	1.4936	0.0022	1.3144
Glutathione	1.1354	0.0048	1.2642
Creatine	1.3278	0.0286	1.0478
Carnitine	0.8020	0.0331	1.2326
Methionine	1.1709	0.0484	1.0852

FDR: False Discovery Rate; VIP: variable importance on projection.

**Table 4:** Clinical and anthropometric parameters in premenopausal women with and without depression.

	Pre ND, (21)	Pre D (21)	*p*
Age (years) ^a^	44.3 ± 0.7	44.8 ± 0.8	0.608
BMI (kg/m^2^) ^a^	26.8 ± 1.2	29.3 ± 1.0	0.114
FSH (mUI/dL) ^a^	8.6 ± 1.3	7.0 ± 0.9	0.318
Cortisol (ng/dL) ^a^	6.6 ± 0.8	6.0 ± 0.8	0.620
BFP ^a^	36.1 ± 1.6	40.3 ± 1.5	0.066
Muscle Mass (kg) ^a^	25.2 ± 1.9	31.4 ± 2.0	**0.034**
BDI (score) ^a^	4.7 ± 0.6	18.9 ± 1.7	**<0.000**
Diabetes ^b^	0.0%	4.7%	0.311
Hypertension ^b^	19.0%	14.3%	0.679
Exercise ^b^	57.1%	14.3%	**0.004**

Pre ND: premenopausal women without depression; Post D: premenopausal women with depression. ^a^ Means ± SE, analyzed by Student’s *t* test. ^b^ Percentages analyzed by chi-squared test. BMI: body mass index; BFP: percentage body fat; BDI: Beck Depression Inventory score. Bold values indicate statistical significance.

**Table 5:** Fold-change and variable importance on projection (VIP) of plasma polar metabolites in premenopausal women.

	Fold-Change (D/ND)	VIP
Proline	0.9277	0.9943
Guanosine	1.1811	1.1250
Adenosine	0.9953	0.8699
Hypoxanthine	0.8934	0.5664
4−Hydroxyproline	2.3518 *	1.6441
Lysine	1.1013	1.0905
2−Aminobutyric acid	0.9493	0.5869
Adenosine monophosphate	0.8173	0.4206
Dimethylglycine	1.6595 *	1.4842
Citrulline	0.9509	1.2853
Cystine	1.3541	1.0062
Creatine	1.1235	0.7455
Pantothenic acid	0.7769	0.7956
Aspartic acid	1.1453	0.9636
Isocitric acid	1.1114	0.6755
Carnitine	1.1448	1.1767
Fumaric acid	1.1059	0.5928
Glutamic acid	1.2259	1.3787
Histidine	0.9470	1.3093
Methionine	0.9661	1.1109
Glycine	0.9465	0.8499
Kynurenic Acid	2.0251	1.0403
Arginine	1.1039	0.8819
Succinic acid	1.1559	0.9289
Uridine	1.0621	0.2917
Oxidized glutathione	0.4959 *	2.1168
Glutathione	0.9976	0.5093
Valine	1.0977	1.2988
Isoleucine	1.1195	1.1936
Lactic acid	1.1035	0.8348
Glutamine	1.0049	0.9662
Ornitine	1.1609	0.8215
Phenylalanine	1.1102	1.3965
Tyrosine	1.1284	1.0349
Kynurenine	0.9203	0.8449
Serine	0.9467	0.9429
Asymmetric dimethylarginine	1.0518	0.6525
Asparagine	1.0584	0.9984
Tryptophan	1.0664	0.8062
Alanine	1.0021	0.7768
Cysteine	1.4670	0.8213
Choline	0.9744	0.8032
Creatinine	0.9942	0.8302
Uric acid	1.0402	1.1055
Threonine	0.9791	0.7418
2−Ketoglutaric Acid	1.1100	0.3913
Acetylcarnitine	1.0655	1.2127
Leucine	0.8730	1.1372
TMAO	1.1001	0.3463
Taurine	1.1554	0.6501

D: premenopausal women with depression symptoms; ND: premenopausal women without depression symptoms. * FDR < 0.05.

**Table 6:** Plasma polar metabolites significantly affected in premenopausal depression.

Metabolite Name	Fold-Change (D/ND)	FDR	VIP
Oxidized glutathione	0.4959	0.0137	2.1168
4-Hydroxyproline	2.3518	0.0406	1.6441
Dimethylglycine	1.6595	0.0433	1.4842

FDR: False Discovery Rate; VIP: variable importance on projection.

## Data Availability

The data supporting reported results can be found in the “Metabolomics Workbench” website, accessible through https://www.metabolomicsworkbench.org/ (accessed on 1 February 2024). The Project ID is PR001980, and the doi is 10.21228/M8SJ0J.
